# Supplementary material for: COVID-19 lockdowns and demographically-relevant Google Trends: A cross-national analysis
Source: PLoS One. 2021 Mar 17;16(3):e0248072. doi: 10.1371/journal.pone.0248072 (PMC7968661; doi:10.1371/journal.pone.0248072)
Supplement: S1 Table — (DOCX) [file pone.0248072.s001.docx]

**S1 Table. Search terms by topic, country, and language**

|  | **Language:** | **English** | **French** | **Spanish** | **Italian** | **German** | **German** | **English** | **Spanish (Mexican)** |
| --- | --- | --- | --- | --- | --- | --- | --- | --- | --- |
|  | **Country:** | **United Kingdom** | **France** | **Spain** | **Italy** | **Austria** | **Germany** | **United States** | **United States** |
| **CONTRACEPTION** | **Condom** | Condom | preservatif  préservatif  capote | condon  condón  preservativo | preservativo  profilattico  preservativi  profilattici  goldone  goldoni | kondom  präservativ  gummi | kondom  präservativ  gummi | condom | condón  preservativo |
|  | **Emergency pill** | morning after pill  emergency pill  emergency contraception | pilule lendemain  pillule lendemain | pildora dia despues  pildora dia siguiente | pillola giorno dopo | pille danach | pille danach | morning after pill  emergency pill  emergency contraception | pildora dia siguiente  anticonceptivo de emergencia |
|  | **Pregnancy test** | pregnancy test | test grossesse  test de grossesse | prueba embarazo  test embarazo | test gravidanza | schwangerschaftstest | schwangerschaftstest | pregnancy test | prueba embarazo  test embarazo |
|  | **Abortion** | Abortion | avortement  avorter  ivg | aborto  pastilla abortiva | aborto  abortire  pillola abortiva | abtreibung  schwangerschaftsabbruch  abtreiben | abtreibung  schwangerschaftsabbruch  abtreiben | abortion | aborto  pastilla abortiva |
| **FERTILITY** | **Plan child** | child plan  planning child  child project  birth plan | avoir enfant  projet enfant | planeación familiar  proyecto hijo | progetto figlio  pianificazione bambino | kinderwunsch  Kind haben  Kind bekommen | kinderwunsch  Kind haben  Kind bekommen | child plan  planning child  child project  baby plan  birth plan | planeación familiar |
|  | **Plan other child** | other child  second child  third child | autre enfant  deuxième enfant  second enfant  troisième enfant  troisieme enfant | otro hijo  segundo hijo  tercer hijo | secondo figlio  terzo figlio  altro figlio | zweites kind  drittes kind  weiteres kind  noch ein kind | zweites kind  drittes kind  weiteres kind  noch ein kind | other child  second child  third child | otro hijo  segundo hijo  tercer hijo |

| **COUPLE** | **Wedding** | marriage  wedding  married | mariage  marier | boda  matrimonio | matrimonio  sposarsi | ehe  hochzeit  heirat  Trauung | ehe  hochzeit  heirat  Trauung | marriage  wedding  married | boda  matrimonio  casados |
| --- | --- | --- | --- | --- | --- | --- | --- | --- | --- |
|  | **Dating app** | dating app  dating site  tinder  online dating | site rencontre  tinder | tinder  meetic | sito incontri  app incontri  tinder | tinder  dating app  Singlebörse  Dating Seite | tinder  dating app  Singlebörse  Dating Seite | dating app  dating site  tinder  online dating | Tinder  apps de citas  apps para ligar  noviazgo en línea  citas en línea |
|  | **Relationship** | Relationship | couple | pareja | coppia | beziehung  partnerschaft | beziehung  partnerschaft | relationship | relacion de pareja  pareja |
| **DIVORCE** | **Divorce** | Divorce | divorce | divorcio | divorzio  divorziare | scheidung | scheidung | divorce | divorcio |
|  | **Break up** | end relationship  break-up  breakup  breakup | séparation couple  separation couple  rupture couple | ruptura  separación  fin de la relación | separazione | trennung | trennung | end relationship  break-up  breakup  breakup | ruptura  separación  fin de la relación |
