# Supplementary material for: COVID-19 lockdowns and demographically-relevant Google Trends: A cross-national analysis
Source: PLoS One. 2021 Mar 17;16(3):e0248072. doi: 10.1371/journal.pone.0248072 (PMC7968661; doi:10.1371/journal.pone.0248072)
Supplement: S2 Table — (DOCX) [file pone.0248072.s002.docx]

| S2 Table: Lockdown dates in European countries and U.S. states | | | | |
| --- | --- | --- | --- | --- |
| European Countries | | | | |
| Austria | 16-Mar-20 | Italy | 9-Mar-20 | |
| France | 17-Mar-20 | Spain | 14-Mar-20 | |
| Germany | 22-Mar-20 | UK | 23-Mar-20 | |
| United States | | | | |
| Alabama | 4-Apr-20 | Montana | 28-Mar-20 | |
| Alaska | 28-Mar-20 | Nebraska | -- | |
| Arkansas | -- | Nevada | 1-Apr-20 | |
| Arizona | 31-Mar-20 | New Hampshire | 27-Mar-20 | |
| California | 19-Mar-20 | New Jersey | 21-Mar-20 | |
| Colorado | 26-Mar-20 | New Mexico | 23-Mar-20 | |
| Connecticut | 23-Mar-20 | New York | 22-Mar-20 | |
| District of Columbia | 1-Apr-20 | North Carolina | 30-Mar-20 | |
| Delaware | 24-Mar-20 | North Dakota | -- | |
| Florida | 3-Apr-20 | Ohio | 23-Mar-20 | |
| Georgia | 3-Apr-20 | Oklahoma | -- | |
| Hawaii | 25-Mar-20 | Oregon | 23-Mar-20 | |
| Iowa | -- | Pennsylvania | 1-Apr-20 | |
| idaho | 25-Mar-20 | Rhode Island | 28-Mar-20 | |
| Illinois | 21-Mar-20 | South Carolina | 7-Apr-20 | |
| Indiana | 24-Mar-20 | South Dakota | -- | |
| Kansas | 30-Mar-20 | Tennessee | 31-Mar-20 | |
| Kentucky | 26-Mar-20 | Texas | 2-Apr-20 | |
| Louisiana | 23-Mar-20 | Utah | -- | |
| Maine | 2-Apr-20 | Vermont | 25-Mar-20 | |
| Maryland | 30-Mar-20 | Virginia | 30-Mar-20 | |
| Massachusetts | 24-Mar-20 | Washington | 23-Mar-20 | |
| Michigan | 24-Mar-20 | West Virginia | 24-Mar-20 | |
| Minnesota | 27-Mar-20 | Wisconsin | 24-Mar-20 | |
| Mississippi | 31-Mar-20 | Wyoming | -- | |
| Missouri | 6-Apr-20 |  |  | |
| Note: Only state-wide lockdown dates reported for the United States. Local restrictions are not reported. | | | |  |
|  | | | |  |
