# Supplementary material for: COVID-19 lockdowns and demographically-relevant Google Trends: A cross-national analysis
Source: PLoS One. 2021 Mar 17;16(3):e0248072. doi: 10.1371/journal.pone.0248072 (PMC7968661; doi:10.1371/journal.pone.0248072)
Supplement: S3 Table — (DOCX) [file pone.0248072.s003.docx]

**S3 Table. Outlier search results excluded based on public interest events driving searches**

|  |  | **UK** | **France** | **Spain** | **Italy** | **Austria** | **Germany** | **US (english)** | **US (Spanish)** |
| --- | --- | --- | --- | --- | --- | --- | --- | --- | --- |
| CONTRACEPTION | Condom |  | ● 2016-11-13 : Truman Capote movie on Arte TV |  |  |  |  | ● 2018-04-01 : Snorting condom challenge |  |
|  | Emergency pill | ● 2017-07-16 : Controversy with Boots refusing to cut the price of morning after pill |  |  |  |  |  |  |  |
|  | Pregnancy test |  |  |  |  |  |  |  |  |
|  | Abortion | ● 2017-01-22 : “Mexico City Policy” (executive order) ● 2018-05-20 : referendum about abortion in Ireland ● 2019-05-12 : Human Life Protection Act (Alabama abortion law) | ● 2017-01-22 : “Mexico City Policy” (executive order) ● 2018-05-20 : referendum about abortion in Ireland ● 2019-05-12 : Human Life Protection Act (Alabama abortion law) | ● 2017-01-22 : “Mexico City Policy” (executive order) ● 2018-08-05 : Rejection of legal abortion in Argentina ● 2018-05-20 : referendum about abortion in Ireland ● 2019-05-12 : Human Life Protection Act (Alabama abortion law) | ● 2017-01-22 : “Mexico City Policy” (executive order) ● 2018-05-20 : referendum about abortion in Ireland ● 2019-05-12 : Human Life Protection Act (Alabama abortion law) | ● 2017-01-22 : “Mexico City Policy” (executive order) ● 2018-05-20 : referendum about abortion in Ireland ● 2019-05-12 : Human Life Protection Act (Alabama abortion law) | ● 2017-01-22 : “Mexico City Policy” (executive order) ● 2018-05-20 : referendum about abortion in Ireland ● 2019-05-12 : Human Life Protection Act (Alabama abortion law) | ● 2016-10-16 : 3rd USA presidential debate (“late-term abortion”) ● 2017-01-22 : “Mexico City Policy” (executive order) ● 2019-01-20 : Reproductive Health Act (NY abortion law) ● 2019-01-27: Reproductive Health Act (NY abortion law) ● 2019-02-03: Reproductive Health Act (NY abortion law) ● 2019-05-12 : Human Life Protection Act (Alabama abortion law) ● 2019-05-19 : Human Life Protection Act (Alabama abortion law) |  |
| FERTILITY | Plan Child |  |  |  | ● 2017-12-17 : Son of Elena Santarelli ill of cancer |  |  |  |  |
|  | Plan other child | ● 2017-09-03 : K. Middleton pregnancy ● 2018-04-22 : K. Middleton childbirth | ● 2017-09-03 : K. Middleton pregnancy ● 2018-04-22 : K. Middleton childbirth | ● 2017-09-03 : K. Middleton pregnancy ● 2018-03-18 : Third child of Pilar Rubio ● 2018-03-25 : Third child of Pilar Rubio ● 2018-04-22 : K. Middleton childbirth | ● 2017-09-03 : K. Middleton pregnancy ● 2018-04-22 : K. Middleton childbirth ● 2018-10-28 : Measure about giving state lands to people having a third child | ● 2017-09-03 : K. Middleton pregnancy ● 2018-04-22 : K. Middleton childbirth | ● 2017-09-03 : K. Middleton pregnancy ● 2018-04-22 : K. Middleton childbirth |  |  |
| COUPLE | Wedding | ● 2018-05-13 : Wedding Markle-Harry ● 2018-05-20 : Wedding Markle-Harry | ● 2018-05-13 : Wedding Markle-Harry ● 2018-05-20 : Wedding Markle-Harry | ● 2018-05-13 : Wedding Markle-Harry ● 2018-05-20 : Wedding Markle-Harry ● 2019-06-16 : Wedding Belén Esteban | ● 2018-05-13 : Wedding Markle-Harry ● 2018-05-20 : Wedding Markle-Harry | ● 2018-05-13 : Wedding Markle-Harry ● 2018-05-20 : Wedding Markle-Harry | ● 2018-05-13 : Wedding Markle-Harry ● 2018-05-20 : Wedding Markle-Harry | ● 2018-05-13 : Wedding Markle-Harry ● 2018-05-20 : Wedding Markle-Harry | ● 2018-02-04 : Movie “La boda de Valentina” ● 2018-02-1: Movie “La boda de Valentina” ● 2019-06-30 : Boda de Carmen Villalobos |
|  | Dating |  |  |  |  |  |  |  |  |
|  | Relationship |  |  |  |  |  |  |  |  |
| DIVORCE | Divorce | ● 2016-09-18 : Divorce Jolie-Pitt | ● 2016-09-18 : Divorce Jolie-Pitt | ● 2016-09-18 : Divorce Jolie-Pitt | ● 2016-09-18 : Divorce Jolie-Pitt ● 2017-05-07 : Supreme court decision about divorce law | ● 2016-09-18 : Divorce A. Jolie-B. Pitt | ● 2016-09-18 : Divorce Jolie-Pitt | ● 2016-09-18 : Divorce Jolie-Pitt |  |
|  | Breakup |  |  |  | ● 2016-02-14 : Music “Nessun grado di separazione” |  | ● 2016-10-23 : Love scandal around Sarah Lombardi | ● 2018-06-03 : Liza Koshy-David Dobrick break-up (Youtube stars) |  |
