# Supplementary material for: COVID-19 lockdowns and demographically-relevant Google Trends: A cross-national analysis
Source: PLoS One. 2021 Mar 17;16(3):e0248072. doi: 10.1371/journal.pone.0248072 (PMC7968661; doi:10.1371/journal.pone.0248072)
Supplement: S4 Table — (DOCX) [file pone.0248072.s004.docx]

S4 Table: Event Study estimates for lockdown-, family planning-, and fertility related search terms, European countries

|  | Lockdown | Condom | Emergency pill | Pregnancy test | Abortion | Plan Child | Plan other children |
| --- | --- | --- | --- | --- | --- | --- | --- |
|  | b/se | b/se | b/se | b/se | b/se | b/se | b/se |
| T-6 | ref. | ref. | ref. | ref. | ref. | ref. | ref. |
| T-5 | 0.11 | -0.02 | -0.06 | -0.00 | -0.01 | 0.25 | 0.03 |
|  | (0.08) | (0.03) | (0.07) | (0.06) | (0.09) | (0.56) | (0.06) |
| T-4 | 0.20 | -0.04 | -0.06 | -0.04 | -0.10* | 0.04 | -0.10 |
|  | (0.13) | (0.04) | (0.04) | (0.02) | (0.03) | (0.07) | (0.09) |
| T-3 | 0.90 | 0.03 | 0.01 | -0.04 | 0.05 | 0.09 | -0.09 |
|  | (0.51) | (0.04) | (0.03) | (0.03) | (0.10) | (0.04) | (0.05) |
| T-2 | 4.06 | 0.02 | -0.08 | -0.09* | -0.08** | -0.18 | -0.14 |
|  | (2.22) | (0.04) | (0.11) | (0.04) | (0.02) | (0.15) | (0.11) |
| T-1 | 11.81 | -0.03 | -0.06 | -0.16* | -0.08* | 0.13 | -0.21 |
|  | (4.79) | (0.03) | (0.06) | (0.06) | (0.03) | (0.43) | (0.12) |
| T 0 | 46.04* | -0.15* | -0.34** | -0.29** | -0.12 | 0.02 | -0.22 |
|  | (15.55) | (0.05) | (0.08) | (0.05) | (0.05) | (0.48) | (0.11) |
| T 1 | 26.83** | -0.13 | -0.49*** | -0.22*** | -0.07 | -0.21* | -0.24** |
|  | (4.23) | (0.07) | (0.05) | (0.03) | (0.06) | (0.08) | (0.06) |
| T 2 | 20.23*** | -0.04 | -0.52*** | -0.09 | -0.13* | -0.26 | -0.19 |
|  | (1.77) | (0.14) | (0.05) | (0.08) | (0.05) | (0.20) | (0.09) |
| T 3 | 20.81*** | -0.02 | -0.47** | -0.19** | -0.05 | 0.38 | -0.23** |
|  | (2.68) | (0.12) | (0.07) | (0.04) | (0.06) | (0.50) | (0.05) |
| T 4 | 18.39*** | -0.08 | -0.46** | -0.11 | -0.02 | -0.26 | -0.00 |
|  | (2.36) | (0.13) | (0.07) | (0.05) | (0.06) | (0.20) | (0.11) |
| T 5 | 16.77** | -0.01 | -0.51*** | -0.09 | -0.06 | 0.10 | 0.06 |
|  | (3.01) | (0.15) | (0.05) | (0.04) | (0.04) | (0.29) | (0.11) |
| T 6 | 16.72** | -0.00 | -0.48*** | -0.10 | -0.11* | 0.10 | -0.07 |
|  | (2.51) | (0.12) | (0.06) | (0.05) | (0.03) | (0.21) | (0.14) |
| T 7 | 12.71** | -0.03 | -0.47*** | -0.00 | -0.07 | 0.00 | -0.17 |
|  | (2.71) | (0.08) | (0.06) | (0.04) | (0.04) | (0.09) | (0.15) |
| T 8 | 8.59** | -0.03 | -0.33** | -0.12** | -0.08 | 0.85 | 0.10 |
|  | (2.06) | (0.08) | (0.06) | (0.02) | (0.06) | (0.81) | (0.14) |
| T 9 | 7.01* | -0.00 | -0.17** | -0.04 | -0.16 | 0.13 | -0.02 |
|  | (1.86) | (0.07) | (0.03) | (0.06) | (0.07) | (0.15) | (0.10) |
| T 10 | 5.08** | 0.03 | -0.21* | -0.01 | -0.15** | 0.20 | 0.10 |
|  | (0.94) | (0.05) | (0.08) | (0.03) | (0.03) | (0.09) | (0.12) |
| T 11 | 3.89** | 0.16 | -0.10 | 0.02 | -0.16** | 0.61 | 0.00 |
|  | (0.96) | (0.11) | (0.06) | (0.04) | (0.03) | (0.42) | (0.10) |
| T 12 | 3.35* | 0.02 | -0.09 | 0.01 | -0.13 | 1.07 | 0.01 |
|  | (0.87) | (0.05) | (0.06) | (0.04) | (0.05) | (0.85) | (0.08) |
| T 13 | 3.29** | 0.05 | -0.03 | 0.09* | 0.05 | 0.38 | 0.15 |
|  | (0.52) | (0.03) | (0.05) | (0.03) | (0.08) | (0.19) | (0.07) |
| Observations | 1404 | 1404 | 1404 | 1404 | 1404 | 1404 | 1404 |

Note: Google Trends extraction made July 6, 2020. All models include controls for country-specific public events with implications for specific searches (see Appendix Table A3).

* p<.05, ** p<.01, *** p<.001.
