# Supplementary material for: COVID-19 lockdowns and demographically-relevant Google Trends: A cross-national analysis
Source: PLoS One. 2021 Mar 17;16(3):e0248072. doi: 10.1371/journal.pone.0248072 (PMC7968661; doi:10.1371/journal.pone.0248072)
Supplement: S5 Table — (DOCX) [file pone.0248072.s005.docx]

S5 Table. Event Study estimates for lockdown-, family planning-, and fertility related search terms, United States

|  | Lockdown | Condom | Emergency pill | Pregnancy test | Abortion | Plan Child | Plan other children |
| --- | --- | --- | --- | --- | --- | --- | --- |
|  | b/se | b/se | b/se | b/se | b/se | b/se | b/se |
| T-6 | ref. | ref. | ref. | ref. | ref. | ref. | ref. |
| T-5 | 0.00 | -0.00 | -0.05 | 0.02 | -0.03 | -0.31 | 0.06 |
|  | (0.05) | (0.04) | (0.09) | (0.03) | (0.03) | (0.17) | (0.07) |
| T-4 | 0.37** | 0.05 | 0.01 | -0.02 | -0.00 | -0.11 | -0.07 |
|  | (0.11) | (0.05) | (0.10) | (0.04) | (0.03) | (0.18) | (0.08) |
| T-3 | 1.97** | 0.06 | 0.10 | -0.04 | 0.05 | -0.09 | -0.10 |
|  | (0.56) | (0.04) | (0.12) | (0.03) | (0.03) | (0.16) | (0.07) |
| T-2 | 8.14*** | 0.03 | -0.03 | -0.07* | 0.05 | -0.20 | -0.15 |
|  | (1.24) | (0.04) | (0.12) | (0.03) | (0.04) | (0.19) | (0.08) |
| T-1 | 19.64*** | 0.04 | -0.03 | -0.11*** | -0.02 | -0.15 | -0.16* |
|  | (1.61) | (0.05) | (0.12) | (0.03) | (0.03) | (0.15) | (0.07) |
| T 0 | 32.64*** | -0.05 | -0.12 | -0.10** | -0.06 | -0.23* | -0.26*** |
|  | (1.60) | (0.04) | (0.10) | (0.03) | (0.04) | (0.11) | (0.07) |
| T 1 | 11.43*** | -0.03 | -0.11 | -0.05 | -0.19*** | -0.29* | -0.11 |
|  | (0.51) | (0.04) | (0.09) | (0.03) | (0.04) | (0.12) | (0.06) |
| T 2 | 9.12*** | -0.08 | -0.25** | -0.15*** | -0.18*** | -0.25 | -0.11 |
|  | (0.49) | (0.04) | (0.07) | (0.03) | (0.03) | (0.13) | (0.07) |
| T 3 | 8.92*** | -0.11*** | -0.23* | -0.12*** | -0.12** | -0.27 | -0.09 |
|  | (0.57) | (0.03) | (0.09) | (0.03) | (0.04) | (0.15) | (0.08) |
| T 4 | 10.34*** | -0.16*** | -0.20* | -0.07** | -0.08 | 0.21 | 0.18 |
|  | (0.79) | (0.04) | (0.08) | (0.03) | (0.05) | (0.28) | (0.11) |
| T 5 | 9.92*** | -0.15*** | -0.26*** | -0.07* | -0.20*** | 0.07 | -0.03 |
|  | (1.15) | (0.04) | (0.04) | (0.03) | (0.05) | (0.16) | (0.07) |
| T 6 | 6.95*** | -0.12** | -0.06 | -0.03 | -0.28*** | -0.27 | 0.25* |
|  | (0.54) | (0.04) | (0.11) | (0.03) | (0.03) | (0.14) | (0.12) |
| T 7 | 6.34*** | -0.04 | -0.22* | -0.04 | -0.21*** | 0.09 | 0.14 |
|  | (0.70) | (0.04) | (0.10) | (0.03) | (0.03) | (0.22) | (0.09) |
| T 8 | 4.28*** | -0.14*** | -0.28** | -0.02 | -0.30*** | 0.07 | 0.29*** |
|  | (0.56) | (0.04) | (0.10) | (0.04) | (0.03) | (0.19) | (0.07) |
| T 9 | 2.72*** | -0.21*** | -0.36*** | -0.05 | -0.32*** | -0.20 | 0.23** |
|  | (0.26) | (0.05) | (0.09) | (0.04) | (0.03) | (0.12) | (0.07) |
| T 10 | 1.99*** | -0.10* | -0.19* | -0.01 | -0.22*** | 0.11 | 0.03 |
|  | (0.25) | (0.04) | (0.09) | (0.02) | (0.03) | (0.12) | (0.09) |
| T 11 | 1.45*** | -0.13** | 0.03 | -0.01 | -0.07 | -0.27 | 0.09 |
|  | (0.14) | (0.04) | (0.12) | (0.03) | (0.04) | (0.16) | (0.08) |
| T 12 | 1.35*** | -0.04 | -0.22* | 0.02 | -0.03 | -0.14 | 0.01 |
|  | (0.18) | (0.05) | (0.10) | (0.04) | (0.03) | (0.18) | (0.09) |
| T 13 | 1.14*** | -0.11* | -0.22 | 0.08* | -0.04 | -0.06 | 0.02 |
|  | (0.14) | (0.05) | (0.11) | (0.03) | (0.03) | (0.19) | (0.08) |
| Observations | 10062 | 10062 | 9360 | 10062 | 10062 | 10062 | 10062 |

Note: Google Trends extraction made July 6, 2020. All models include controls for country-specific public events with implications for specific searches (see Appendix Table A3).

* p<.05, ** p<.01, *** p<.001.
