# Supplementary material for: COVID-19 lockdowns and demographically-relevant Google Trends: A cross-national analysis
Source: PLoS One. 2021 Mar 17;16(3):e0248072. doi: 10.1371/journal.pone.0248072 (PMC7968661; doi:10.1371/journal.pone.0248072)
Supplement: S6 Table. Event study estimates for union formation- and union dissolution-related terms, European countries — (DOCX) [file pone.0248072.s006.docx]

S6 Table: Event Study estimates for union formation- and union dissolution-related terms, European countries

|  | Dating app | Relationship | Wedding | Break up | Divorce |
| --- | --- | --- | --- | --- | --- |
|  | b/se | b/se | b/se | b/se | b/se |
| T-6 | ref. | ref. | ref. | ref. | ref. |
| T-5 | -0.20 | -0.01 | -0.04 | 0.06 | 0.03 |
|  | (0.11) | (0.05) | (0.02) | (0.09) | (0.03) |
| T-4 | -0.11 | 0.03 | -0.01 | -0.10 | 0.04 |
|  | (0.06) | (0.02) | (0.04) | (0.04) | (0.05) |
| T-3 | -0.19 | -0.02 | -0.05 | -0.07 | 0.00 |
|  | (0.08) | (0.04) | (0.02) | (0.04) | (0.07) |
| T-2 | -0.21 | -0.07 | -0.11** | -0.12 | -0.09 |
|  | (0.10) | (0.04) | (0.03) | (0.07) | (0.05) |
| T-1 | -0.28* | -0.15* | -0.19* | -0.20* | -0.16* |
|  | (0.10) | (0.05) | (0.06) | (0.07) | (0.04) |
| T 0 | -0.42* | -0.12* | -0.42*** | -0.37** | -0.31* |
|  | (0.13) | (0.05) | (0.04) | (0.09) | (0.09) |
| T 1 | -0.35* | -0.00 | -0.43*** | -0.15 | -0.32** |
|  | (0.13) | (0.06) | (0.02) | (0.14) | (0.06) |
| T 2 | -0.39 | 0.02 | -0.41*** | -0.13 | -0.34* |
|  | (0.15) | (0.06) | (0.02) | (0.09) | (0.09) |
| T 3 | -0.34 | 0.07 | -0.39*** | -0.04 | -0.28* |
|  | (0.16) | (0.05) | (0.04) | (0.09) | (0.10) |
| T 4 | -0.23 | 0.07 | -0.36*** | 0.05 | -0.08 |
|  | (0.13) | (0.06) | (0.02) | (0.11) | (0.12) |
| T 5 | -0.34 | 0.02 | -0.40*** | 0.05 | -0.20* |
|  | (0.13) | (0.07) | (0.04) | (0.11) | (0.05) |
| T 6 | -0.33 | 0.04 | -0.43*** | -0.05 | -0.20* |
|  | (0.14) | (0.03) | (0.02) | (0.09) | (0.08) |
| T 7 | -0.32 | 0.06 | -0.49*** | 0.06 | -0.10 |
|  | (0.14) | (0.04) | (0.06) | (0.16) | (0.04) |
| T 8 | -0.26 | 0.04 | -0.53*** | 0.00 | -0.04 |
|  | (0.15) | (0.02) | (0.04) | (0.07) | (0.04) |
| T 9 | -0.19 | -0.01 | -0.57*** | 0.04 | -0.09 |
|  | (0.15) | (0.07) | (0.06) | (0.09) | (0.04) |
| T 10 | -0.13 | -0.03 | -0.57*** | 0.12 | -0.00 |
|  | (0.14) | (0.05) | (0.06) | (0.11) | (0.11) |
| T 11 | -0.14 | 0.01 | -0.50** | 0.08 | -0.03 |
|  | (0.11) | (0.02) | (0.07) | (0.07) | (0.05) |
| T 12 | -0.13 | 0.03 | -0.45** | 0.01 | -0.00 |
|  | (0.11) | (0.02) | (0.07) | (0.08) | (0.04) |
| T 13 | -0.10 | 0.06 | -0.43* | 0.05 | 0.04 |
|  | (0.11) | (0.03) | (0.12) | (0.09) | (0.04) |
| Observations | 1404 | 1404 | 1404 | 1404 | 1404 |

Note: Google Trends extraction made July 6, 2020. All models include controls for country-specific public events with implications for specific searches (see Appendix Table A3).

* p<.05, ** p<.01, *** p<.001.
