# Supplementary material for: COVID-19 lockdowns and demographically-relevant Google Trends: A cross-national analysis
Source: PLoS One. 2021 Mar 17;16(3):e0248072. doi: 10.1371/journal.pone.0248072 (PMC7968661; doi:10.1371/journal.pone.0248072)
Supplement: S7 Table — (DOCX) [file pone.0248072.s007.docx]

S7 Table. Event Study estimates for union formation- and union dissolution-related terms, United States

|  | Dating app | Relationship | Wedding | Break up | Divorce |
| --- | --- | --- | --- | --- | --- |
|  | b/se | b/se | b/se | b/se | b/se |
| T-6 | ref. | ref. | ref. | ref. | ref. |
| T-5 | 0.01 | 0.00 | -0.02* | -0.06 | 0.00 |
|  | (0.03) | (0.02) | (0.01) | (0.04) | (0.01) |
| T-4 | 0.01 | -0.02 | -0.04*** | -0.07 | -0.02 |
|  | (0.02) | (0.02) | (0.01) | (0.04) | (0.02) |
| T-3 | -0.06** | -0.06** | -0.11*** | -0.06 | -0.07** |
|  | (0.02) | (0.02) | (0.01) | (0.03) | (0.03) |
| T-2 | -0.12*** | -0.13*** | -0.20*** | -0.08 | -0.18*** |
|  | (0.02) | (0.02) | (0.01) | (0.05) | (0.02) |
| T-1 | -0.14*** | -0.15*** | -0.27*** | 0.00 | -0.23*** |
|  | (0.03) | (0.03) | (0.01) | (0.04) | (0.02) |
| T 0 | -0.11*** | -0.06* | -0.28*** | 0.05 | -0.23*** |
|  | (0.02) | (0.03) | (0.01) | (0.04) | (0.02) |
| T 1 | -0.06* | 0.01 | -0.25*** | 0.15*** | -0.14*** |
|  | (0.03) | (0.03) | (0.01) | (0.04) | (0.03) |
| T 2 | -0.05* | 0.04 | -0.21*** | 0.16*** | -0.11*** |
|  | (0.02) | (0.03) | (0.01) | (0.04) | (0.02) |
| T 3 | -0.05 | 0.04 | -0.15*** | 0.11* | -0.11*** |
|  | (0.03) | (0.03) | (0.01) | (0.05) | (0.02) |
| T 4 | -0.07* | 0.08*** | -0.16*** | 0.16*** | -0.08** |
|  | (0.03) | (0.02) | (0.01) | (0.04) | (0.02) |
| T 5 | -0.11** | 0.05* | -0.17*** | 0.11** | -0.08** |
|  | (0.03) | (0.02) | (0.01) | (0.04) | (0.02) |
| T 6 | -0.11*** | 0.05* | -0.19*** | 0.14** | -0.02 |
|  | (0.03) | (0.02) | (0.01) | (0.05) | (0.02) |
| T 7 | -0.10** | 0.03 | -0.18*** | 0.18*** | -0.04 |
|  | (0.03) | (0.03) | (0.01) | (0.04) | (0.03) |
| T 8 | -0.08* | 0.02 | -0.21*** | 0.04 | -0.02 |
|  | (0.03) | (0.02) | (0.01) | (0.04) | (0.02) |
| T 9 | -0.15*** | -0.01 | -0.24*** | 0.05 | -0.01 |
|  | (0.03) | (0.02) | (0.01) | (0.04) | (0.02) |
| T 10 | -0.11** | 0.04 | -0.23*** | -0.03 | 0.05 |
|  | (0.03) | (0.02) | (0.01) | (0.03) | (0.03) |
| T 11 | -0.05 | 0.03 | -0.18*** | 0.05 | 0.08** |
|  | (0.03) | (0.02) | (0.01) | (0.04) | (0.03) |
| T 12 | -0.12*** | 0.02 | -0.18*** | 0.13** | 0.07** |
|  | (0.02) | (0.02) | (0.01) | (0.04) | (0.02) |
| T 13 | -0.06* | 0.01 | -0.17*** | 0.16*** | 0.01 |
|  | (0.03) | (0.03) | (0.01) | (0.04) | (0.03) |
| Observations | 10062 | 10062 | 10062 | 10062 | 10062 |

Note: Google Trends extraction made July 6, 2020. All models include controls for country-specific public events with implications for specific searches (see Appendix Table A3).

* p<.05, ** p<.01, *** p<.001.
