# Supplementary material for: COVID-19 lockdowns and demographically-relevant Google Trends: A cross-national analysis
Source: PLoS One. 2021 Mar 17;16(3):e0248072. doi: 10.1371/journal.pone.0248072 (PMC7968661; doi:10.1371/journal.pone.0248072)

**S1 Fig. Deviation from the mean Google search interest for wedding and marriage related terms by U.S. state (top panel) and European country (bottom panel).**


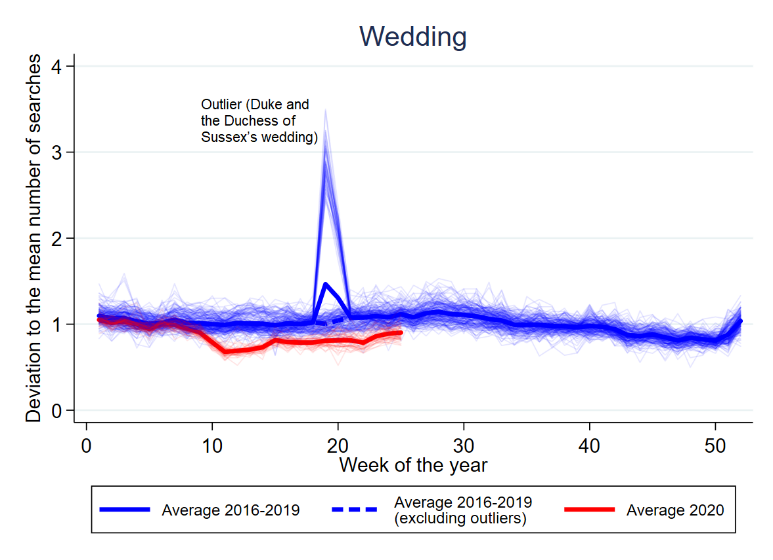


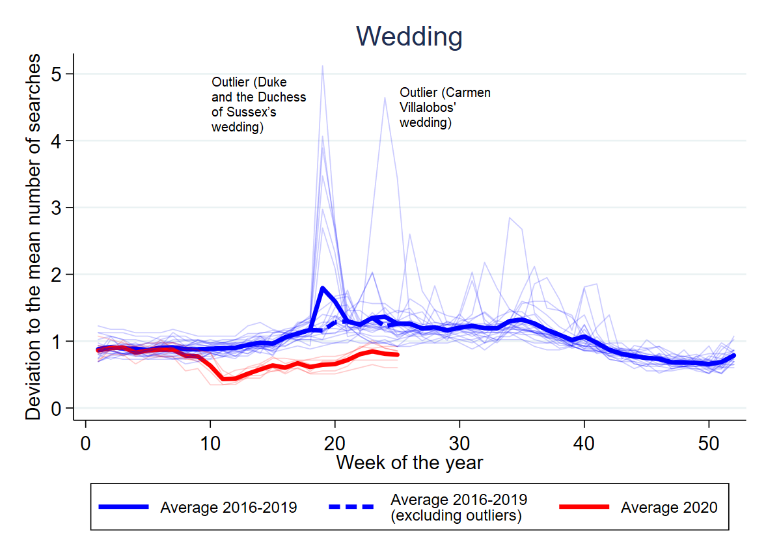

Supplement: S1 Fig — Deviation from the mean Google search interest for wedding and marriage related terms by U.S. state (top panel) and European country (bottom panel). (DOCX) [file pone.0248072.s008.docx]
