# Supplementary material for: COVID-19 lockdowns and demographically-relevant Google Trends: A cross-national analysis
Source: PLoS One. 2021 Mar 17;16(3):e0248072. doi: 10.1371/journal.pone.0248072 (PMC7968661; doi:10.1371/journal.pone.0248072)
Supplement: Attachment — Submitted filename: ResponsetoReviewers_FINAL.docx [file pone.0248072.s009.docx]

We are grateful for the excellent comments from each of the reviewers. Below, we summarize each comment (in italics) and detail our response. We believe the reviews and corresponding revisions have considerably strengthened the manuscript.

**Reviewer #1:**

(1) *There are two types of search functions when using Google Trends: “search term” and “topic”. They usually generate completely different results when using the same keyword. Please explicitly specify which one was used in Section 2.*

We used “search term” rather than “topic” for three reasons, which are now discussed in the Data section of the manuscript. First, we prioritized focusing on terms that were actually entered by Google searchers, rather than on generalized topics. Second, we used translations of each English term, provided by national experts, that were intended to capture country-specific searches targeting the same underlying construct rather than relying on Google’s word-specific translation algorithm. Third, this approach allowed us to assess the adequacy of the term vis-à-vis the underlying construct of interest by assessing the most frequent search requests associated with each term and subsequently excluding terms that returned results that did not reflect the construct of interest. Fourth, we included, in relevant cases, two spellings for the same word, including frequent spelling mistakes, accent or not. We have explained this strategy in the text, including providing examples of ambiguous terms.

(2) *People in non-English speaking countries are also likely to use English search terms, and the likelihood varies across countries. For example, I tested the term “relationship” versus “beziehung”+ “partnerschaft” in Germany and Austria during Jan to Jun 2020, “relationship” accounts for 22% and 24% of its German corresponding words in terms of Google search volumes. However, in France and Spain, it only accounts for 1.5% and 1.6% of its French and Spanish corresponding words. Such differences should not be neglected. Therefore, the authors should explain how do they reduce language bias.*

Non-English-speaking countries in our analysis include Germany, Austria, Spain, Italy, and France. The reviewer makes an excellent point that people in those countries may use English search terms, especially in Germany and Austria. This concern can be broadened to encompass omitted (unexamined) search terms related to a construct, more generally, with non-examination of English terms in non-English speaking countries as a specific example thereof. That is, for any language in each country we may not have identified the full range of commonly used search terms, despite our efforts to be as comprehensive as possible. However, given that our main identification strategies rely on change over time (and not on level) of searches for a given set of terms, these “missing terms” (including English searches) are problematic only if searches for the missing term are differentially affected by the lockdown compared to searches for the synonymous terms included in our study. If this is not the case, the coefficients will not be biased, although they may be less precisely estimated.

In response to this important point, however, we tested the robustness of our results to the addition of the English terms associated with “condom” and “wedding” in analyses of non-English-speaking countries. We chose the term “condom” because the English term “condom” is widely known and unambiguous, and also because the impact of lockdowns on search intensity related to “condom” significantly differed across European countries. We chose “wedding” because this is the search term for which our results are strongest, and also because the term “wedding planner” is often used in non-English speaking countries. Results for condom-related searches differ only slightly when the English term “condom” is added to the list of country-specific search terms. Results on condom-related search intensity remain consistent with those of our primary analyses for Germany with the addition of the English term. The magnitude ty is smaller (though still positive) for Austria when the English term is included. The (negative) impact of lockdown on condom-related searches in France, Spain and Italy is unaffected by inclusion of the English term. When the English term wedding is added to the set of wedding-related search terms, results are unaffected in all countries. These two examples indicate that, while English terms are searched in non-English speaking countries as well, excluding them does not substantially alter our results. We added a paragraph in the section “Preferred model and robustness checks” commenting the robustness of our results to the list of terms used for each search domain.

(3) *I am not sure if the term “event study” is appropriate here. According to Fama, Fisher Jensen and Roll (1969) and Binder (1998), the event study methodology was initially proposed to examine the effect of a stock split announcement on stock prices. The method was later used in many accounting, economic and finance domains. However, the method does not seem to be used in many sociological studies. If the authors can justify it by referencing several seminal articles in sociology/demography used the same term, it would be fine to keep it. Otherwise, it is suggested to use a new phrase.*

“Event study” is recently commonly used in economics to refer to panel econometrics methods that aim to compare outcomes within individuals before and after an event (child birth, widowhood, in our case lockdown). So long as there are no systematic changes in other underlying determinants of the outcome over time, except for timing of the event of focus, differences in trajectories can be interpreted as causal. A paper using this methodology to evaluate air pollution after the burning of firecrackers in India has been recently published in *PLOS ONE* (Ghei, & Sane 2018). For clarity, we now refer to our approach as a “panel event-study design” throughout the manuscript and cite some of the articles below that use the same methodology.

Angelov, N., Johansson, P. and Lindahl, E. 2016. “Parenthood and the Gender Gap in Pay”. Journal of Labor Economics, 34 (3): 545–579.

Bertrand, M., Goldin, C. and Katz, L.F. 2010. "Dynamics of the Gender Gap for Young Professionals in the Financial and Corporate Sectors". American Economic Journal: Applied Economics, 2(3): 228-255, July.

Ghei, D., & Sane, R. (2018). Estimates of air pollution in Delhi from the burning of firecrackers during the festival of Diwali. *PloS one*, *13*(8), e0200371.

Kleven, H. Landais, C. and Søgaard, J.E. 2019 "Children and Gender Inequality: Evidence from Denmark". American Economic Journal: Applied Economics, 11(4): 181-209.

Kleven, H., Landais, C., Posch, J., Steinhauer, A., & Zweimüller, J. 2019. “Child Penalties across Countries: Evidence and Explanations”. AEA Papers and Proceedings, 109 (May): 122-126.

**Reviewer #2:**

(1) *The introductory section offer a succinct and pertinent overview of the expected effects of the pandemic on future demographic trends. However, the authors mention that we can only ‘hypothesize that the pandemic and related lockdowns will have an impact on future family demographic trends’. While this is true, there have been a number of recent studies that have provided evidence on things like fertility intentions, which are in themselves an outcome of interest for family demographers (https://www.demographic-research.org/volumes/vol43/47/). I understand that some of this literature might have been published after the paper was submitted, but acknowledging it will help the motivation of the paper stronger. Note that this is not to say that the literature review is outdated (e.g., the Wilde et al paper is very recent).*

We have added in multiple places in the manuscript (introduction, background, results), reference to Luppi, Arpino, & Rosina (2020) study of fertility intentions during the lockdown. This study is of particular interest because the set of countries is common to ours (with the exception of Austria) but the study uses different data (survey on fertility intentions) and methods. We did not identify other relevant recent research that we have not cited.

Luppi, F., Arpino, B., & Rosina, A. (2020). The impact of COVID-19 on fertility plans in Italy, Germany, France, Spain and UK.

(2) *The authors focus on Europe and the US but do not justify their geographic focus. Given that Google data is, to some extent global, why were only these regions chosen? Up to the end of page four, I had the impression that the authors were giving a global picture of the spread of the pandemic and its consequences for family demography. In the last paragraph of page four, there is a sudden jump to a focus on Europe and the US: “As such, we expect considerable heterogeneity across countries—and, in particular, between Europe and the United States—in the degree to which individuals and families experience economic uncertainty and hardship.” The authors should explain why we would expect particularly high levels of heterogeneity between Europe and the United States? Can we really expect them to vary more compared to other regions of the Global South?*

We now identify the geographical areas of study (U.S. and Europe) at the end of first paragraph of the introduction. Comparing developed countries to the Global South is complicated by large differences in levels of fertility level, contraception prevalence, divorce risk and, perhaps most importantly, internet use patterns (including socioeconomic and educational differences in the populations that do so). As such we believe including middle- and/or lower-income countries is beyond the scope of this article. In addition, as we acknowledge in the manuscript, the selection of European countries was, in part, driven by our ability to access country-specific appropriate translations of our search terms—which could be confirmed by a member of our team or our international colleagues with expertise in family demography. We note (footnote #4) that we had initially intended to include Belgium and Switzerland in our analyses, but ultimately excluded those countries given their bi- to tri-lingual nature which resulted in considerable heterogeneity in search trends, with many language-specific terms failing meet Google Trends’ minimal reporting threshold.

(3) *The assertion that recent “Google search trends are useful for predicting subsequent demographic trends” should be qualified. Wilde et al. (2020) create predictions about future fertility but whether these will turn out to be true or not is an open question at the time of writing. Furthermore, the authors show no evidence of search data actually, and successfully, predicting any demographic trends. This limits the believability of the claim that the claim that web searches are “likely to provide insight into future trends in [demographic] domains”. I suggest including references to nowcasting demographic dynamics using social media data as a base for these claims. The work by Zagheni et al. is particularly useful for this (e.g., https://www.jstor.org/stable/26622775?seq=1#metadata_info_tab_contents) and Billari, who you do cite elsewhere.*

We have qualified the assertion that Google Trends data are useful for predicting demographic trends and have cited the suggested authors (which we had already cited, but elsewhere and for other work). We also cite Choi and Varian’s (2012) analyses using Google searches for predicting economic trends. The specific Zagheni et al. paper suggested by the reviewer (“ Leveraging Facebook's Advertising Platform to Monitor Stocks of Migrants”) focuses on social network data which, while interesting and important, is not directly connected to our analyse; thus we do not cite it in the current paper. However, we do cite Cesare et al. (2018), which is co-authored by Zagheni and focuses on the use of digital traces for demographic research. We also cite Billari et al. (2013) on fertility forecasting with google data.

In addition, the reviewer is correct that our analyses cannot be assumed to predict actual demographic behavior. We deleted all passages that imply such and have revised the final paragraph of the manuscript to make this clear.

Cesare, N., Lee, H., McCormick, T., Spiro, E., & Zagheni, E. (2018). Promises and pitfalls of using digital traces for demographic research. Demography, 55(5), 1979-1999.

Billari, F. C., D’Amuri, F., Marcucci, J. (2013), Forecasting births using google. Paper presented at the annual meeting of the Population Association of America, April, New Orleans, LA.

(4) *In page 5, Data section, the authors mention that the list of search terms is a crucial component of the study. The authors say that they “identified a series of search terms for each construct”. However, it is not clear how these terms were chosen and why they vary so much across languages. The text says that the “translations focused on achieving consistent cultural, linguistic, and in-country meaning rather than direct translation of specific words” but it would be good to know the actual mechanics of how the terms were chose – were in-country experts consulted?*

We have added a description of the motivation and method for our choice of search terms (rather than topics), and the rigor we applied to checking/testing each individual term/set of terms by assessing associated queries, accounting for spelling errors, and validating their translations with native speakers. Nonetheless, we acknowledge that there is an arbitrary aspect to selecting search terms. As noted in our response to Reviewer #1, we may have omitted some relevant search terms despite our efforts to be as comprehensive as possible. However, because our approach is relative (change over time), these “missing terms” have the potential to bias our results if—and only if—searches for the omitted term are affected differently by lockdowns than searches for the synonymous terms that we include. If this is not the case, our coefficients may be less precisely estimated, but will not be biased. We also note that, in preliminary analyses, we tested searches for a much larger set of terms (many focusing on economic issues), but retained only those closely related to demographic behaviors to clearly focus the paper.

(5) *Was the initial keywords list taken from a published study? Considering the central importance of keyword choice it is somewhat surprising that the authors do not provide, as a sensitivity analysis, results for alternative keyword configurations. Do the results change if some keywords are included/excluded? Does it matter that some languages have different number of keywords for each domain (e.g. one word for ‘abortion’ in English and four in German)? The Mexican Spanish “pastilla abortive” is incorrect (it should be “abortiva”).*

Our research team, in consultation with a cross-national group of colleagues, initially identified and tested a much larger range of potential search terms. After testing this list, we culled the terms to select those that were unambiguous, consistently used over time, and sufficiently searched (to avoid series’ with a large number of null values). We also included groups of terms for which there were multiple spellings or ways of saying the same thing in a particular country. The selected words are the result of these exercises. Our methodology is similar to that of Wilde et al. (2020, see page 18 “Word selection methodology”).

It is possible that the number of search terms included for a given country will affect the overall level of searches identified in that country. However, because the number of search terms on which we focus is constant within each country over time, differences therein will not affect relative change over time in searches for *those terms* as a function of lockdowns. As noted in our responses to Reviewer #1 (point #2) and Reviewer #2 (point #4), this may have implications for the precision of our estimates, though not for bias thereof. As noted above, we were as comprehensive and careful as possible in selecting terms. In addition, we conducted robustness checks for several topics by selecting only the most frequently searched term rather than the larger list of terms. The panel event-study results are robust to this approach and the conclusions reached do not change. Although we observe some variation in the magnitude of our estimates, their directions remain unchanged. Moreover, any changes in magnitude do not appear to be systematic. For instance, we find a stronger negative impact of lockdown on search intensity for “condom” but a weaker negative impact of lockdown on search terms for “emergency pill” in Europe when we include only a single search term. In both cases, as in the case of other terms, the overall pattern of results is consistent with that from our primary specifications.

Unsurprisingly, our country-by-country difference-in-difference estimates are more sensitive to the inclusion/exclusion of search terms. For instance, the positive impact of lockdown on search terms associated with “condom” is weaker in Germany and Austria when only one term is retained. The negative impact of lockdown on search intensity for words associated with “divorce” tends to be stronger in both European countries and in the U.S., while the positive impact on searches related to “break up” tends to be stronger, especially in the U.S., when using only one term. Again, however, these differences are found in the magnitude but not direction of the effects. We have added a paragraph “Preferred model and robustness checks” section commenting the robustness of our results to the list of words for each search terms.

Finally, the reviewer is correct that the Mexican Spanish for abortion is “pastilla abortiva”, not “pastilla abortive”. This was a typo in the manuscript, but not in the actual search term used. We have corrected the typo.

(6) *The authors state that “In order to observe the potential impact of lockdown policies on search trends, we also collected country- and state-specific lockdown dates as well as data on COVID-19 case, death, and recovery rates”. It would be good to know the source of these data and their granularity (e.g. weekly, sub-national). This is important because, in page 10, the authors mention that they use these data to calibrate their models. E.g., “First, we controlled for the number of confirmed COVID cases or the number of COVID related-deaths”.*

We have cited the sources of the COVID tracking data, which are available on a daily basis from <https://coronavirus.jhu.edu/map.html> and <https://github.com/CSSEGISandData/COVID-19> for each U.S. state and the European countries considered. We extracted weekly data.

(7) *From a demographic perspective, it would be interesting to know why the authors use Covid cases/deaths as an indicator of pandemic lethality, rather than the more commonly-used excess death measure, which is available on a weekly basis from the HMD STMF: https://www.mortality.org/. Similarly, it is unclear if the authors used weekly data on Covid cases/deaths. One is led to believe so given statements like: “Controlling for the number of cases tended to reduce the magnitude of the estimates. However, this also reflects that the number of confirmed cases was higher in the period closely proceeding lockdowns and in the subsequent weeks” but this should be explicit.*

We used weekly data on COVID cases or COVID-related deaths, which we tested in separate models. We now justify why we focus on cases and deaths. Specifically, we focus on these measures because they are explicitly and frequently reported by the mainstream media such that they are likely to influence individuals’ perceptions of the seriousness of the pandemic and, thereby, influence individuals’ behaviors by affecting related stress and worry. These controls are not intended as scientific measures of the pandemic (in which case excess deaths would be more relevant). Rather, they are intended to reflect information widely communicated via mainstream sources. Which strategy is preferable is, of course, debatable. However, given that our results are not sensitive to the inclusion of cases or deaths it is unlikely that they would be sensitive to excess mortality, which also is likely less known to average citizens.

(8) *Table 2 in the Appendix has lockdown dates, which presumably are the dates of the start of lockdowns. There are no dates for the lifting or changing of the policies on restrictions on movement. Countries introduced different restrictions in the time considered, relaxing and constraining measures over time. Similarly, lockdowns in Europe became more localised over time (i.e., going from national- to regional-level). I believe that the Oxford COVID-19 Government Response Tracker has been keeping track of these data but confess that I have not used these data personally. This has implications for the statistical analyses as well: it seems that lockdowns are treated as binary outcomes in model 1 (i.e., t indicates time to/from lockdown start). The same applies for the diff-in-diff model. You state that “we now include a single binary variable which is coded “1” during the period in which a lockdown is in effect, denoted as 1{ ≥ 0}, rather than the indicators for weeks preceding of following a lockdown.” Does this account for the changing nature of lockdowns over time? If so, it would be could to make this explicit. Please clarify how, and if, you account for these nuanced in your measurement of lockdowns. If you do not account for this, please also mention it as a limitation of the research.*

We have clarified that we focus on initiation of a state or country’s first lockdown and do not account for length, stringency, or enforcement of the lockdown. We code a state or country as experiencing lockdown in the first week of its first lockdown and in all weeks thereafter for two reasons. First, we are interested in identifying the average effect of any lockdown (stay at home order) in various contexts. Whereas lockdown implementation occurred in most developed countries (states) regardless of other contextual factors (e.g., social policies), the length and stringency thereof is likely jointly determined with other policies and pandemic response initiatives such that it is likely endogenous.

We added the following paragraph to the text to better define how we measure lockdowns: “The policies implemented by different countries to slow the spread of COVID-19 were quite diverse; initially, the most common and strongest restrictions were stay-at-home orders. Abouk and Heydari (52) demonstrate that the initiation of “lockdown” restrictions had a strong causal impact on reduced social interactions. Because demographic behaviors are strongly linked to social interactions, we define the beginning of the lockdown by the first stay-at-home order at the state (in U.S.) or country (in Europe) level. ”

Second, our empirical strategy (panel event study and difference-in-differences) is optimal for use with a binary treatment. Thus, we select all states or countries which implemented a lockdown as our treated group (and ignore other restrictions and characteristics of the lockdown). Note that our panel-event study does not rely on states that never initiated a lockdown (all countries in our European analyses initiated a lockdown) but takes advantage of the variation in the timing of lockdown initiation (across states) for identification. In contrast, the difference-in-differences analyses include states that did and did not initiate lockdowns and are identified by pre-post variation between treatment and control states (or by pre-post variation within European countries). As such, the two empirical strategies complement each other.

We acknowledge that this strategy is unable to distinguish effects of lockdown length and stringency, as well as subnational differences within the European countries (which should be rare given that, for most European countries, the lockdown was very quickly implemented at the national level—unlike in the U.S.). Additionally, while lockdown initiations are clearly demarcated, lifting of lockdowns in some instances was progressive and, thus, fuzzy, making it difficult to specify an exact end date. We thus focus on lockdown implementation, which we argue is the event that is most likely to have generated demographically-relevant concerns. We discuss these important issues in the limitations section.

(9) *I am generally convinced by the standardised index of search interest proposed by the authors. However, I would appreciate it if the authors could elaborate on what they mean by “It can be shown that ̃ = ̅̅̅ , where ̅ is the average index of relative searches for a term in country c over the January 2016-June 2020 period.” There is no need to include this in the text, I just want to make sure that I understand what they mean by this last statement.*

Google Trends provides an index of *relative* search interest for a given term in which the series is normalized by dividing all data points by the maximum value of the series (in a given location and time period). As such, Google Trends always provides a series with a maximum value equal to 100. There is no intuitive way to directly compare Google Trends series’ between different countries because of this normalization. We transform the Google Trends series index by dividing all data points by the average relative search interest.

The proof for the passage of concern (“It can be shown that $\tilde{R}_{cyw}=\frac{S_{cyw}}{\bar{S_{c}}}$, where $\bar{S_{c}}$ is the average index of relative searches for a term in country *c* over the January 2016-June 2020 period.”) is:

$$\tilde{R}_{cyw}= \frac{R_{cyw}}{\bar{R}_{c}}= \frac{\frac{S_{cyw}}{S_{c}^{m}}}{\frac{\bar{S}_{c}}{S_{c}^{m}}}= \frac{S_{cyw}}{\bar{S_{c}}}$$

This indicates that the transformed series $\tilde{R}_{cyw}$ represents searches for a given term at a given time in a country relative to the average searches interest for the term in the country of a given time period. To state it differently, we measure how searches at a given time compare to searches in “normal” (counterfactual) times. Our transformed series $\tilde{R}_{cyw}$ is easy to interpret and comparable across countries because the coefficients represent deviations from the mean.

(10) *It is unclear how search outliers were identified. From the discussion in page 9, it appears that outliers were deduced from the data (e.g., Fig 1 in Appendix). However, there were presumably many ‘smaller’ outliers that were not so evident and might have biased the data (e.g., subnational scandals or events not in English). The last paragraph in page 10 suggest that this might have been the case. The last robustness check was, according to the authors, skewed by an (presumably not previously identified) abortion-related debate associated with the “Human Life Protection Act” in the US. Can we expect similar mini-scandals to also skew Google Trends data systematically? Was there any systematic attempt to identify potential outliers using other data sources?*

Outliers were selected on a purely empirical basis from the observed data. We were alerted to the presence of outliers by identifying variation in search patterns that, upon investigation, appeared to be associated with local events that were *a priori* unknown to us. Of course, thresholds for defining outliers can be somewhat arbitrary. In the revised manuscript, we have specified that selection of outliers was *empirically* driven and specified our approach to cross-validating their identification. Specifically, to understand potential outliers and their determinants, we analyzed internet searches in specific locations that were suggestive of questionable patterns. Appendix Table A3 gives the list of these outliers, some of which are country specific. For instance, Belén Esteban’s wedding during the week of the 16^th^ of June 2019 is associated with a surge of search interest for “wedding” in Spain only. Another interesting example is that the movie “Truman Capote” was on TV in France during the week of the 13^th^ of November 2016. Given that “capote” means condom in French, we observe a surge in search interest for “capote” on the relevant week in France. By investigating the results for “capote” searches in France in that week, we were able to identify that the surge should not be interpreted as a sudden interest in condoms, but was rather related to the movie.

(11) *In Table 1, why is the number of observations only reported for some regions (e.g., not included for France)? It would be useful to know how you treated missing values (i.e., country/week combinations for which Google returned no results). In the footnote of Table 1 for example, you mention that “Spanish searchers in the U.S. returned null searches in all states except California, Florida, New York, and Texas.”. However, the table provides betas for all domains, except for Emergency pill and Plan Child. Can you please explain the logic behind this and how to interpret these coefficients given the high number of missing values?*

The number of observations was, indeed, missing from the table for European countries when reported separately. This was a mistake; we thank the reviewer for pointing it out. Regarding missing values, we distinguished a missing series from missing values within a series. When search intensity is very low for a term, Google Trends returns missing values. For some terms, typically in small population areas, search activity was so low that Google Trends returned missing values for almost all weeks. This is the case for Spanish words for most states in the U.S., except for the four larges Hispanic population States (California, Florida, New York, and Texas). We considered a series “missing” when Google Trends returned missing values for most weeks and, thus, discarded the relevant states or countries for this term in the relevant analysis. For some series, Google Trends returned non-missing values for almost all weeks, with a few exceptions. In this case, we considered that the missing values was a signal for very low search intensity in the particular week (but not in most weeks) and coded as zero. For Spanish terms in the U.S., only California, Florida, New York and Texas returned non-missing results over (almost) all state/week combinations. Therefore, the results must be interpreted on these four states only and it do not necessarily represent Spanish speakers from other states. We have not included this level of detail in the current manuscript in describing out treatment of missing data, but are happy to do so at the editor’s request.

(12) *In the introduction, the authors hypothesize that lockdowns will lead to fewer demographic consequences in countries that better managed the economic fallout from the pandemic and provided a stronger financial safety net. However, the paper never returns to this hypothesis. If it is central to the study, it should be discussed. If it is not, the authors may want to reconsider whether it should be stated at all.*

We now return to this hypothesis at the start of the Discussion section and highlight it throughout our conclusion.

(13) *The authors need to clarify the mechanisms linking search behaviour to future fertility. The paper assumes that changes in search behaviour may be an indicator of future fertility behaviour but do not, in my opinion, provide ant credible evidence to that respect. Admittedly, doing so is without the scope of this paper, but the authors could refer to other studies that have indeed established a robust link between, for example, searches for ‘family planning’ and fertility nine months later in other contexts (ie, pre-covid). Otherwise, claims like: “Notably, however, we do observe a significant 18 percent increase in such searches in France in the post-lockdown period. This may mean that lockdown will have positive effects on fertility in France, which has particularly generous family policies” are less credible than they could be. The same is true of the concluding statement that “Overall, our results do not suggest that there will be a broad impact of national lockdowns on future family demography in European countries”.*

We now provide a brief discussion of the extent to which search behavior may reflect underlying worries or concerns, as well as the extent to which it has been linked in prior research to future demographic and economic behaviors, in the Introduction section. In addition, we have removed speculation on future actual demographic behaviors noting only that such will need to be empirically examined in future research (see also response to Reviewer #2, point #3, above).

(14) *Related to the last point, I would suggest that the authors do not generalise their results beyond the observed evidence. This applies to the conclusion that: “In sum, using Google Trends data, we find evidence that pandemic-induced lockdowns may have small and likely short-lived impacts on future family formation and dissolution behaviours, particularly in the U.S., but are unlikely to influence subsequent fertility.” The authors acknowledge in page 18 that “Google searches do not directly measure behaviors. Future research based on actual levels of demographic behaviors is needed to corroborate whether and how such searches may predict trends in family demography.” As a result, it seems unusual to be so conclusive regarding future demographic behaviour extrapolated from search terms alone.*

As noted above, we have removed such speculation from the manuscript.

(15) *Other comments:*

- *The Reference section does not list all cited paper. Please review this and make sure that all sources are included; e.g., Perrotta et al. (2020).*

We have ensured that the reference list now includes all cited papers.

- *I suggest proof-reading the document to remove spelling errors and typos, e.g.,
  o “Compared to Europe, returns to average search levels were less evident for the U.S. where, even 2 to 3 months after lockdowns were introduced”
  o “Google Trends provides data the relative search index = / only”)
  o “Spanish searchers in the U.S. returned null searches in all states except California, Florida, New York, and Texas.. “*

We have carefully proof-read the paper to correct spelling and typos.

**Reviewer #3**:

(1) *This study uses a valuable data source – Google Trends – to study the impact of the COVID-19 pandemic on union formation/dissolution and fertility. One limitation of Google Trends is that we cannot account individuals’ search intentions (e.g. are individuals searching for ‘wedding’ because they are planning a wedding, or because they are curious about how the pandemic has impacted weddings?). However, Google Trends does capture, given what we know about internet use rates from the World Bank [1] and the popularity of Google as a search engine [2], a broad swath of the population. It is also a source of longitudinal data, and is thus an excellent tool for monitoring attitudes pre- or post-exogenous shock.*

We are grateful for this useful information and the accompanying citations, which we have incorporated in the text of the Introduction, including footnote #1.

(2) *A number of the authors’ findings have face validity when framed in the context of an acute social crisis. However, the fact that searches for divorce increase slightly immediately after lockdown and beyond is surprising, and does not correlate with my expectations given suggestions about economic uncertainty and union dissolution. To me, this unusual finding highlights this study’s need for explicit hypothesis identification at the onset. The authors’ introduction provides a thorough analysis of literature regarding union formation/dissolution and fertility during times of crisis, and the discussion provides anticipated justification for observed trends. However, because there are so many moving parts associated with this study I would encourage the authors to explicitly state a.) what associations they plan to analyze and b.) precisely how these relationships are expected to behave given contextual factors such as location or outbreak severity prior to jumping into their analysis.*

We now include this information in the Introduction, noting that some expectations are *a priori* ambiguous.

(3) *I would also encourage the authors to be careful about framing this analysis in relation to past analyses of exogenous shocks on fertility and union formation. They suggest that overall interest in union formation/dissolution and fertility has not consistently changed across contexts, and may not change in the future. However, I think this is a premature statement given the widespread economic impact of the lockdown in areas with limited government support, and I would refrain from speculating until post-event data is available.*

We clarify in the Conclusion section that our results relate to the period around the (spring 2020) lockdowns and do not capture long term trends relating to, for example, a post-pandemic economic recession. Furthermore, we clarify that our data capture only search interest and cannot be assumed to predict future demographic behavior (see response to Reviewer #2).

(4) *One concern I have with this paper lies in the ambiguity of the term “lockdown,” and selection of a post-lockdown period across contexts. A number of contexts, including many states in the United States, have consistently upheld public health related restrictions that would reasonably inhibit individuals’ ability to leave their homes and/or interact with others. Not only would the duration of these quasi-lockdown states have the potential to extend post-event drops in interest regarding acute fertility concerns (e.g. condoms, abortion, etc.), they could result in greater economic downturns and a lack of consumer confidence, thus shaping demographic trends for years to come. The difference in search volume for relationship/dating terms post-lockdown in Europe versus the United States is a good example of how variability in lockdown measures may impact choices, as travel and recreation remains severely limited in many states well into the latter half of 2020. A more explicit definition of what constitutes lockdown measures may help unpack this trend.*

We have clarified that we focus on initiation of a state’s or country’s first lockdown and do not account for length, stringency, or enforcement of the lockdown (see response to Reviewer #2, point #2 above). In addition, we note that, while COVID distancing policies were quite diverse, the most common and strongest restriction was some period lockdown via stay-at-home order. Abouk and Heydari (2020) show that stay-at-home orders has been the policies that have had the strongest causal impact on reducing social interactions. Because demographic behaviors are strongly linked to social interactions, we define the beginning of the lockdown by the first stay-at-home order at the state or country level.

As discussed in our response to Reviewer #2 (point #2) above, we are unable to distinguish the differential enforcement of lockdowns and do not account for length of initial lockdown or any subsequent lockdowns that may have occurred in a country. Again, we now address this important point (and other related issues) in the Conclusion section.

(5) *Finally, while the authors state that they interpret observed trends within the context of social conditions and economic hardship across contexts, it may be helpful to identify and control for economic impact within their models. As existing literature on fertility and union formation suggests, economic stability is an essential driver in the decision to couple/separate and/or have children. While we have yet to observe the long-term macroeconomic impact of the pandemic, there may be ways to measure perceived economic uncertainty – such as job losses or gains per month - that could provide insight into observed trends.*

We conducted a robustness check of the role of social conditions related to economic hardship by adding a control for Google searches for “unemployment” in the given country/state and week to our models. The figure below shows how lockdowns affected search intensity for the word “unemployment”. We observe a strong impact in the U.S., and heterogeneous impacts across European countries, suggesting stronger economic uncertainty in the U.S. We thus expected our U.S. results to be more highly affected by controlling for “unemployment” than our European results.

As discussed in the “Preferred model and robustness checks” section, controlling for search intensity for “unemployment” changes some of our U.S. estimates, while our results for Europe are essentially unaffected.

For the U.S., our results suggest that the negative impact of the lockdown on fertility-related searches (condom, pregnancy test, abortion) seems to be related to economic uncertainty. In addition, lockdowns seem to have a positive impact on searches related to divorce and family-planning for other children once perceived economic uncertainty has been controlled for in the U.S. We mentioned these two changing results in the text. Full results are only available as supplemental analyses rather than as our primary analyses because the paper is focused on estimating the full effect of lockdown on demographically-relevant searches rather than estimating this effect net of potential underlying mechanisms (e.g., economic uncertainty).
