# Supplementary material for: COVID-19 lockdowns and demographically-relevant Google Trends: A cross-national analysis
Source: PLoS One. 2021 Mar 17;16(3):e0248072. doi: 10.1371/journal.pone.0248072 (PMC7968661; doi:10.1371/journal.pone.0248072)
Supplement: Attachment — Submitted filename: PLOS_searchVolume_Feb2021.docx [file pone.0248072.s010.docx]

The authors have made changes to the discussion of this manuscript that address my concerns regarding the ambiguity of the term “lockdown” and what this means for their finding. Lockdowns do vary in intensity, and it may be difficult to quantify this restriction. I still have reservations about lockdown severity and compliance, but this may lie beyond this scope of this study.

I did not note any significant changes to the discussion of outlier identification. Overall, the discussion of term identification and data cleaning does not seem replicable for other researchers interested in exploring this topic. If replicability is a challenge in the context of analyses that use search data, I would encourage the authors to be transparent about this.

My primary concern with the first iteration of this study concerned how the authors established hypotheses and clarified links between search behavior, fertility/family planning concerns, economic uncertainty and lockdown restrictions. I have similar concerns with this iteration of the paper. The introduction cites that lockdown may result in increased union dissolution as couples deal with increased stress, or it may result in more relationship stability as couples spend more time together. Fertility may increase as the couples have more time to focus on their families and relationship. However, the uncertainty of the pandemic may also reduce fertility rates. The economic fallout of the pandemic and the strictness of lockdown and physical distancing mandate are expected to serve as an important mediating factors that may produce variation in search results across contexts. Overall, it is clear that there are a number of factors that may shape fertility and union concerns, and that we can identify at least a couple of mechanisms underlying anticipated changes.

The results of this study focus primarily on whether demographically relevant keyword searches increase or decrease across countries. What seems to be missing are consistent links between the proposed mechanisms shaping fertility and union formation/dissolution concerns across contexts, anticipated changes in search volume across country context, and confirmation or refutation of anticipated search trends. The authors may consider adding refining their hypotheses by classifying countries studied according to the severity of lockdown restrictions and economic impact they experienced, and establishing anticipated fertility and/or union dissolution trends for each economic uncertainty and/or lockdown intensity category. While it is difficult to fully anticipate how these factors may impact demographic choices given the influence of additional potential control factors and individual agency, explicitly structured hypotheses may help link together sections of this study.

The authors have already taken a step toward this by controlling for unemployment in their models. In their conclusion, they state: “[we] hypothesized that consequences for demographic behaviors might be more marked in countries with less well managed outbreaks and with fewer safety nets, exacerbating feelings of uncertainty and economic insecurity.” They indeed find interesting patterns regarding searches for divorce across contexts, when controlling for unemployment search volume. Accounting for this potential mechanism provides an interesting story, and allows us to do more than simply observe how possible fertility and union formation/dissolution interest may have increased or decreased at the start of the pandemic.

Overall, this study illustrates excellent use of a novel data source, and identifies very interesting patterns in demographically relevant searches pre and post-lockdown. However, I think more work needs to be done to render observed patterns interpretable and grounded in anticipated mechanisms known to drive fertility and union dissolution trends. Accounting for unemployment is a strong start in this direction, but establishing more straightforward, mechanism-focused hypotheses may help.
